# Changes of Oxytocin and Serotonin Values in Dialysis Patients after Animal Assisted Activities (AAAs) with a Dog—A Preliminary Study

**DOI:** 10.3390/ani9080526

**Published:** 2019-08-03

**Authors:** Lucia Francesca Menna, Antonio Santaniello, Alessia Amato, Giuseppe Ceparano, Annamaria Di Maggio, Mario Sansone, Pietro Formisano, Ilaria Cimmino, Giuseppe Perruolo, Alessandro Fioretti

**Affiliations:** 1 Departments of Veterinary Medicine and Animal Productions, Federico II University of Naples, 80134 Naples, Italy; 2 Kidney Dialysis Center Napoli, 80126 Naples, Italy; 3 ASL Napoli1 Centro-CRIUV, 80145 Napoli, Italy; 4 Electrical Engineering and Information Technology Federico II University of Naples, 80125 Naples, Italy; 5 Department of Translational Medical Sciences, Federico II University of Naples, 80131 Naples, Italy

**Keywords:** dog-assisted therapy, animal-assisted activities (AAAs), serotonin, social cooperation, oxytocin, dialysis, dog co-therapist

## Abstract

**Simple Summary:**

This study aimed to improve the moment of dialysis because the emotional management of a person during treatment can help to reduce stress, anxiety and depression. This process positively affects the acceptance and progress of treatment and improves the self-management of the disease, a very important achievement in chronic kidney disease. Serotonin and oxytocin are important neuromodulators of different human behaviours, such as affectivity and socialization, and are involved in the control of stress, anxiety and social cooperation. The relationship between humans and domestic animals provides psychophysical well-being and can facilitate interpersonal bonds by favouring mechanisms involved in social relations. Dogs due to their ethological characteristics, allow the establishment of an active relationship through play, communication and interaction. Animal-assisted activities (AAAs) are structured interventions aimed at improving the psychophysical conditions of people in stressful conditions. Our study was aimed at determining the circulating levels of serotonin and oxytocin in patients who participated in an AAAs program with a dog during dialysis treatment.

**Abstract:**

Our study aimed to measure the levels of serotonin and oxytocin in patients affected by end-stage renal disease (ESRD), undergoing dialysis and participating in a program of animal-assisted activities (AAAs) with a dog. Ten patients with comparable levels of ESRD were enrolled. A blood sample was taken before the start of the study in order to establish basal levels. Eleven meetings were held once a week for 3 months during the last hour of dialysis, and blood samples were collected before and after AAAs. Two more meetings, one month apart from each other, were held two months later without the dog but with the same veterinarian zootherapist. Blood was drawn at the beginning and at the end of each meeting. The samples were then processed for the measurement of serotonin and oxytocin, and data obtained were analysed using analysis of variance with mixed effect models. The results show an increasing level of both serotonin and oxytocin between subsequent meetings with the dog and an increasing trend of inter-intervention levels. Overall, the results suggest that AAAs lead to modifications of serotonin and oxytocin levels, which are also accompanied by behavioural changes of patients.

## 1. Introduction

Animal Assisted Activities (AAIs) have been increasingly used both in health care and in the school context. In fact, they have been divided in Animal Assisted Therapy (AAT), Animal Assisted Activity (AAA) and Animal Assisted Education (AAE) [1]. Humans, like other mammals, develop a necessary bond of attachment that conditions their way of relating. Furthermore, emotional ties and attachment in relationships between people and different animal species have been studied extensively [2,3,4]. The dog is the main species involved in AAIs [5]. This species has an important competence, which is the ability to read the non-verbal language of humans, and moreover, it has developed the interest towards humans being more than other species [6].

In recent years, the effects of AAI have been subjected to a quantitative review [7] and systematic reviews, including randomized studies from 1990 to 2012 [8]. Further studies on the benefits of neurorehabilitation were published from 2001 to 2012 [9] and others related to biopsychosocial results in hospitalized children and adults [10,11,12,13]. Recently, the international scientific literature has shown the large beneficial effects of AAT involving dogs in a wide range of people with problems of psychophysical and mental health, such as adults with Autism Spectrum Disorder [14], as well as adults with Alzheimer disease and other dementias [15,16,17], but also in psychotherapy for adolescents [18]. Particularly regarding dementia care, pet robots have also been used to evaluate their effects on patients [19].

Moreover, this relationship appears to have an important effect of chronic and acute pain. Brown et al. 2003 [20] evaluated only human social support, while later studies showed the effects of the relationship with dogs [21], more precisely, in the medication pain after the replacement of an articulation [22], in pediatric pain [23], and on perceived pain in patients with spinal cord injuries [24].

Finally, these beneficial effects were also assessed in TAA with the involvement of other animal species such as horses for post-traumatic stress disorder in veterans [25,26] and for young people at risk [27].

End-stage renal disease (ESRD) is a major cause of morbidity and mortality worldwide. Due to the lack of potential kidney donors and patient comorbidities, dialysis is the main therapeutic option offered to such patients [28]. Hemodialysis is considered a stressful treatment for patients, although it is essential for their survival. Person-centered care is being promoted more forcefully, allowing patients to improve the management of their own treatment rather than being passive recipients of care [29]. In the UK, the Health System in 2012 focused its interest on customer-centered care, promoting personal care planning, self-management and decision-making shared between patients and healthcare professionals [30]. The involvement of patients and attention to the person are fundamental processes for the acceptance of their state [31]. Feroze et al. [32] showed that patients suffering from chronic kidney disease and undergoing hemodialysis treatment often suffered from depression and anxiety, which have a significant negative influence on the self-management of their disease and lead to the worsening of their condition [33]. Furthermore, depression is often associated with poor quality of life in these patients [34].

These data prompted us to intervene with an AAAs program with a dog to make the time spent during dialysis more enjoyable and thus improve the well-being of the person and the socialization between patients during this phase of the treatment. To our knowledge, this is the first report of such an approach in this therapeutic context.

To assess the effect of the AAAs, ESRD patient serum serotonin and oxytocin levels were measured. Serotonin (5-HT) and Oxytocin (OXT) are important modulators of different behavioural patterns, including human affection and socialization. These chemical messengers interact in the regulation of emotion-based behaviour. The studies highlight the role of regulation of affiliated behaviours and the role of oxytocin for disorders such as autism and other disorders considered social and stress-related, including social phobia, post-traumatic stress disorder and some personality disorders [35,36,37]. Furthermore, studies conducted on 15q dup mice [38] have indicated that interaction of serotonin-oxytocin through 5-HT1A receptors may play a critical role in the normal development of social behaviour. This has led to hypotheses of novel therapeutic strategies in different pathologies, including ASD (autism). Thus, our study aimed to measure the levels of serotonin and oxytocin in patients who participated in an AAAs program with dogs during dialysis treatment.

## 2. Materials and Methods 

### 2.1. Patients

A total of 10 patients (7 men and 3 women) aged between 30 and 50 years with a comparable stage of renal damage and relational difficulties were enrolled in the study. All the patients were chosen by the Kidney Center’s Physician and Psychologist. The inclusion criteria were clinical record (loss of function of both kidneys over 70%, renal parameters: creatinine of about 8–11 mg/dL and azotemia 40–150 mg/dL; threshold values: creatinine: 1.1–1.2 mg/dL, Azotemia: 20–30 mg/dL), treatment cycle (3 times a week during 3 h per session), day and time of treatment, homogeneous socio-demographic characteristics (cultural level, education, family background, absence of a dog at home, similar lifestyle), relational difficulty (the important tendency to isolate themselves and to remain silent, with little possibility of dialogue between them or with the medical staff). During the intervention, through a participatory observation, the psychologist monitored the increase/decrease of the patients’ dialogic and behavioural interactions between themselves, the operators involved, and the dog. The exclusion criteria were refusal to participate in the project, allergy, fear of the dog, animal possession at home, other levels of renal damage, concomitant diseases.

Before they participated, all the patients gave and signed their informed consent for inclusion in the study and to be video-recorded (ISO 9001-2015 Cert. n. 317jSGQ10).

### 2.2. Animal-Assisted Activities (AAAs)

AAAs were performed from January to April 2018. They were held once a week for a total of 11 meetings, lasted about 1 h, and were always performed at the same time, during the last hour of dialysis. The initial 10 min involved reintroducing the dog to each patient, the next 20 min involved structured activity with the dog, a 20-min interview of each patient, and the last 10 min involved the same ending activities each time (Table 1). For the entire duration of the intervention, the dog remained an active part of the study, whether it was actively acting with the play [39,40], or at rest conditions on the dog mat. Its presence in the setting allowed the creation of a relational thrust between operator and patient and facilitated the establishment of a relationship of trust. Two months after this cycle of meetings, two further meetings were held, 4 weeks apart from each other, with the same zootherapist veterinarian and without the dog, who performed similar interventions of play and interviews. All the sessions of AAAs were video recorded.

### 2.3. AAA Team

The work team consisted of professionals trained according to interdisciplinary principles. The Psychologist and Zootherapist Veterinarian, experts in interspecific relationship, worked in team with an animal in situations of human illness. This is a very complex job that requires competence and responsibility. We considered it appropriate for the reference training model to be of a systemic relational approach [41]. The psychotherapist had the competence to establish the most appropriate approach for patients in the setting with the dog, the veterinarian had the competence to help the relationship, but also the ability to recognize the communicative signals of the dog in real time. The vet was in charge of animal health and zoonoses, checked the suitability of the setting, and created play with the dog [16,17,18,19,20,21,22,23,24,25,26,27,28,29,30,31,32,33,34,35,36,37,38,39,40,41,42].

### 2.4. Choice of the Dog

A sterilized 6-year-old male Labrador who had a good score on extroversion, focus motivation, and friendliness, was chosen following the canine Monash personality test [43]. This test was useful to highlight the dog’s relational abilities. Ley et al. [44] showed his affidability. Recently, a meta-analysis demonstrated that this measure, along with behaviour, showed high reliability over time [45,46,47]. The dog was educated together with the conducting veterinarian, who was also the owner, to develop a harmonious and balanced relationship between them. We gave importance to the relational competence of the dog and to the relationship with the veterinarian who was also the owner. From the literature consulted [48,49] it emerged that the dog identified a difference between the owner and other people and improved itsperformance. Even the studies of Pratoprevide and other authors [50,51,52,53] reported that in the presence of its owner, the dog showed greater security towards new stimuli. A harmonic interspecific relationship is one in which the human referent represents the safe base of the dog according to the principles of secure attachment, [2,3,4] and in which the dog does not respond to the need to replace the human being. This is a relationship in which reciprocity is accomplished. This “*reciprocity*” is what occurs between two people, two things, or two groups so that an action or a thing received from one of the two *terms* corresponds to an action or an equivalent thing received from the other *term* [54]. It is therefore a very complex system of relational feedback that start from bodily gestures and attitudes, with the activation of emotional sense-motor models between the two species. This type of relational dimension gives the dog an important sense of security to be able to express itself and neutralize the stress of the first meetings until the next adaptation [3,49,50,51,52,53,54,55]. The veterinary conductor allowed the dog to move away and back even during the session [56]. These conditions are also important for total safety from possible zoonotic risks [42,43,44,45,46,47,48,49,50,51,52,53,54,55,56,57]. The training program followed the guidelines of the national center for sports education (CSEN Rome, Italia). All the exercises were performed to teach the veterinarian to recognize the dog’s communicative behavioural signals. The training of the dog was intended to enable it to manage frustration with self-control and reinforce the permits adapted to the context of the setting The coping times were measured to optimize training [58] and reach a condition in which the dog expressed behavioural pleasure signals (olfactory patrol, (without freezing), no demand, regular breathing, no stress signals (licking, trembling, muscle stiffness, etc.) [59]. All the necessary procedures were undertaken to guarantee a high standard of animal welfare. The dog was monitored on the health level [60] throughout the study cycle.

#### Behavioural and Infective Safety of the Dog

At T1 and T2 of each AAA session, disinfectant wipes (chlorhexidine, TRIS-EDTA, zinc gluconate and glycerin) were used to clean the coat, the fingertips, and the tail of the dog to avoid the transmission of zoonotic agents (e.g., bacteria, fungi, parasitic elements) [60,61,62,63,64,65]. When the dog fidgeted (for example, screaming, slapping) and did not respond to attempts to redirect its behaviour, the veterinarian intervened promptly to minimize the effects of the stressful situation by changing the activity, maintaining a distance between the dog and the stressful stimulus or putting the dog in resting condition on the mat with an “oral discharge” [16].

### 2.5. Blood Sampling and Analysis

At the beginning of the study, a blood sample (5 mL) was taken at the last hour of dialysis for each patient in order to establish the basal levels of oxytocin and serotonin. For all 11 sessions of AAAs with the dog and the last two without the dog, blood samples (5 mL) were taken before each intervention (T1) and immediately after (T2). The study was performed according to the Declaration of Helsinki, and the protocol was approved by the Federico II University Ethical Committee.

Blood samples (5 mL) were collected from the ports used for haemodialysis in tubes for serum preparation, which were centrifuged at 1000× *g* at room temperature for 15 min, 15–30 min after collection. Serum was obtained and deep-frozen (at −80 °C) until processed as a batch for determination of serotonin and oxytocin levels.

The ELISA diagnostic kits used for serotonin and oxytocin are based on a competitive procedure (Enzolife Diagnostics). Assays were performed according to the manufacturer’s instructions. Briefly, patient serum was added to a microwell plate and incubated with serotonin or oxytocin conjugated with alkaline phosphatase for 2 h at room temperature. Then, anti-serotonin or anti-oxytocin antibodies are added for further incubation (for serotonin 2 h at room temperature; for oxytocin 18 h at 4 C), followed by washing to remove the unbound antigen. Next, pNpp was added for 1h at room temperature as a substrate for the alkaline phosphatase and generated a colorimetric reaction which was read in a photometer at the wavelength of 405 nm after the reaction was stopped. Quantification of samples was obtained by comparing the optical density of samples with a master curve prepared by using known standards. The amount of signal was indirectly proportional to the amount of serotonin or oxytocin in the sample.

### 2.6. Statistical Analysis

With the aim to test for within-session changes, we used the Wilcoxon paired test (due to non-normality of data) between T1 and T2. A repeated measures strategy was used to assess the effect of time (independent variable) on the concentrations of serotonin and oxytocin (dependent variables) before and after the AAAs meetings. Because of missing data (39 observations out of 280), balanced one-way ANOVA was not adequate; therefore, linear mixed effects models (LME) were used [66]. With the aim to investigate the return to basal levels, we performed an analysis of variance between the first and the last two sessions.

## 3. Results

### Statistical Analysis

**Figure 1:**
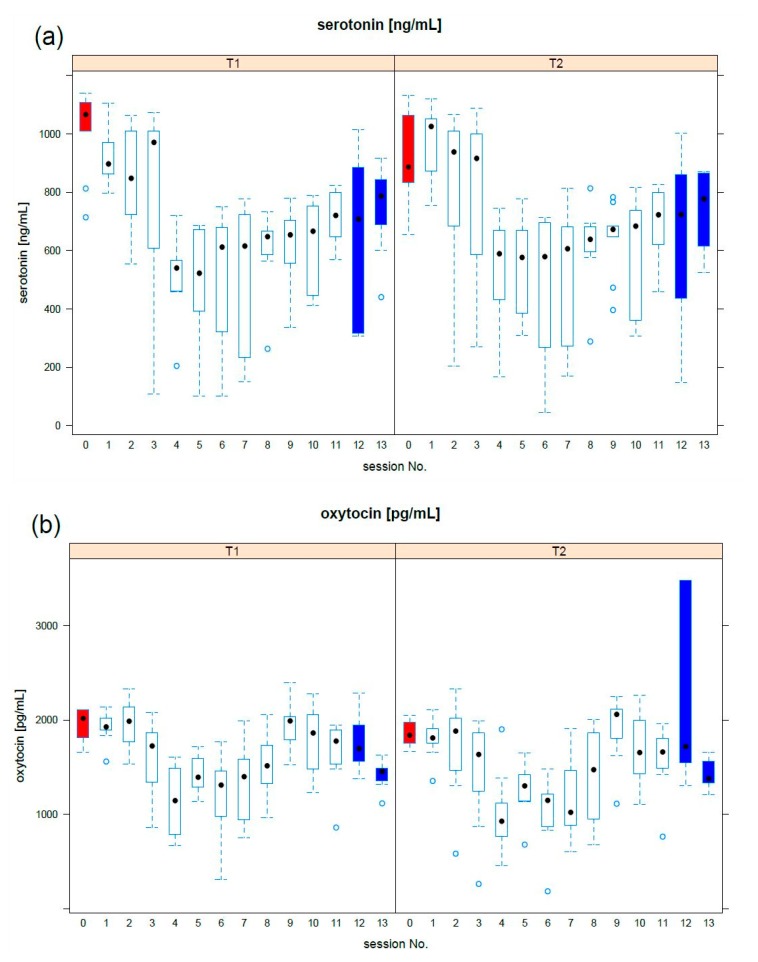
Mean profiles response at T1 and T2: the boxplot of (**a**) serotonin, (**b**) oxytocin was reported per each meeting. The preliminary meeting (red) established basal levels, while the last two meetings (blue) provided information on the long-period effect. Median values are indicated by a black dot.

A statistical analysis was conducted in R [67]. For all the tests, a *p*-value of 0.05 was considered significant. The mean response profile to the activities was bi-phasic for serotonin and oxytocin (Figure 1). In particular, serotonin concentration quickly decreased in the first phase (up to the 4th meeting) and slowly increases in the second phase. Oxytocin showed a comparable behaviour.

**Figure 2:**
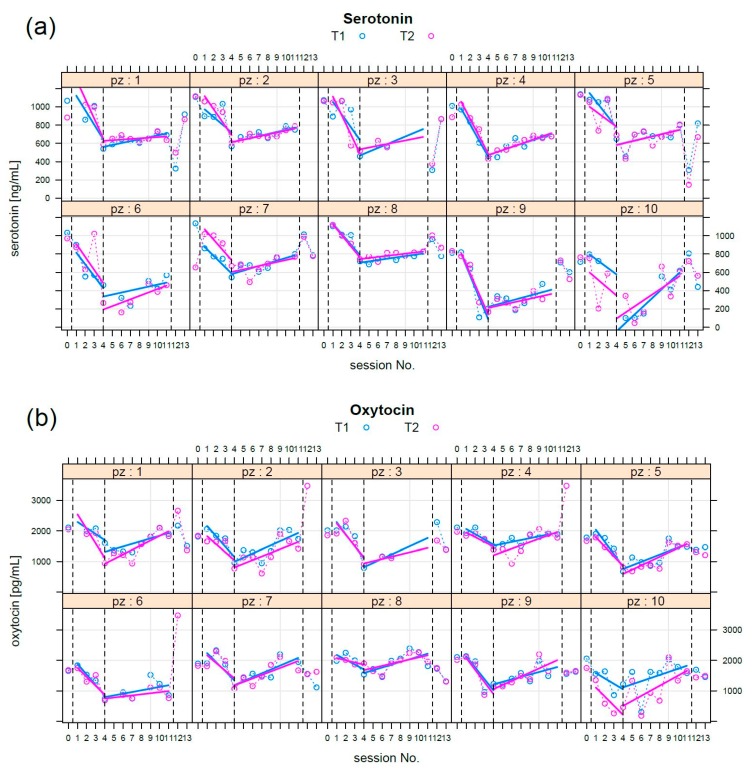
(**a**) Serotonin: per each patient (pz) measurements were reported at T1 (blue circles) and T2 (magenta circles). The first blood sampling had the objective to establish basal levels. The time course of the serotonin level has a clear biphasic shape. During the first phase (sessions 1–4), concentration decreased; during the second phase (sessions 4–11), the level increased. The last two session took place six months apart from session 11, without the dog. Regression lines were superimposed in opportune colours. The vertical dotted lines separate the phases. (**b**) Oxytocin, a biphasic pattern also emerges in this case: the same considerations stated above for serotonin can be repeated here.

For each session, no significant differences (Wilcoxon paired test) were found between T0 and T1 serotonin concentration. The time course of the concentration of serotonin showed a clear biphasic behaviour (Figure 2). Data inspection suggested a biphasic behaviour from the first to the eleventh session with dog and we tested the hypothesis of no change versus a biphasic behaviour. For each phase, we used LME modelling; in particular, three models were compared: first, a patient-dependent horizontal line, second, a patient-dependent (random) intercept and a (fixed) single slope, third, both intercept and a patient-dependent slope.

For the first phase, there was no clear evidence that both random intercept and slope fitted data were better than the fixed slope and the random intercept (AIC = 493, BIC = 499, *p* < 0.05): fixed slope = −135 (ng/mL)/week, fixed intercept = 1103 ng/mL, standard deviation of random effect = 150 ng/mL.

For the second phase, the best explanation (AIC = 832, BIC = 845, *p* < 0.05) was that from both the random intercept and slope models, the fixed intercept = 327 ng/mL, the fixed slope = 31.4 (ng/mL)/week; the standard deviation of random intercept = 275 ng/mL, and the random slope = 17.3 (ng/mL)/week.

We tested for correlation between slopes and age; moreover, we tested for slope differences between men and women (Wilcoxon) and no significant association (*p* > 0.05) was found in both cases.

**Figure 3:**
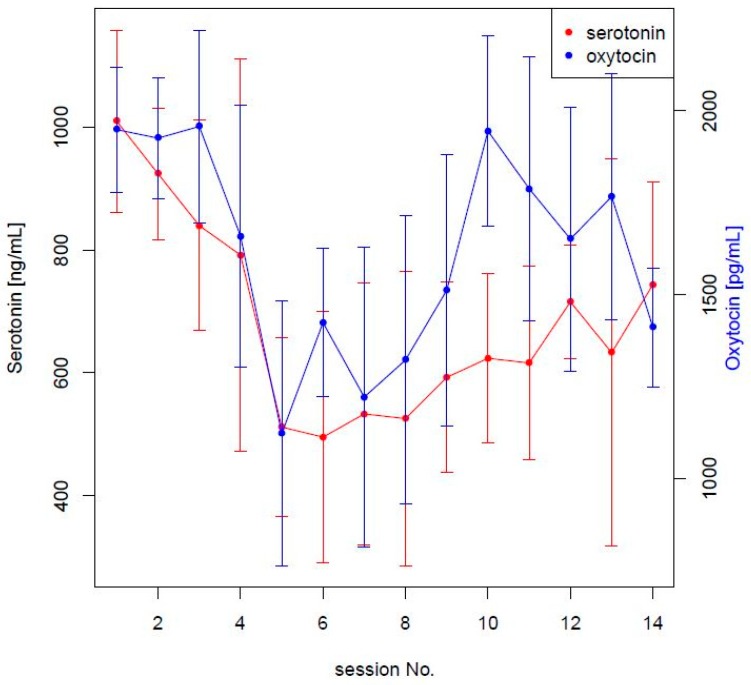
Standard deviations at each session were reported as vertical lines. Only serotonin and oxytocin were reported on different scales in order to appreciate a common biphasic pattern.

In Figure 3, a common biphasic pattern of serotonin and oxytocin is emphasized.

The ANOVA test between the first and the last two sessions revealed a significant difference in both serotonin/oxytocin levels. No significant correlation between slope and age or gender was found.

## 4. Discussion

From these preliminary results, we observed that AAA with a dog can induce the release of serotonin and oxytocin. Significant hormonal movement occurred between one session and another, while the intra-intervention session presented many individual variables to be assessed as significant for the group. It is worth highlighting that this study was not a conventional treatment vs control study but a single group study; as a matter of fact, as detailed in [68], single group studies can be performed in ‘pilot’ longitudinal cohort studies when an implicit comparison with respect to the lack of treatment is known. As there is no evidence in the literature that serotonin and oxytocin levels should change in time because of dialysis, our implicit hypothesis was that serotonin and oxytocin levels would remain bounded by within-subject variability. Therefore, we estimated a basal-level of variability and each subject was considered as the ‘control’ of itself: variations of serotonin and oxytocin were observed with respect to basal-levels. However, this improvement only applies to the time of work because, in the checks that were carried out in the following months, serotonin and oxytocin levels settled approximately on the initial values. The results display an interesting trend also evident in the behaviour of the patients who had been chosen through the behavioural observations of the psychologist who reported an important tendency to isolate themselves and to remain silent, with little possibility of dialogue between themselves or with the medical staff. In fact, patient sociability increased in the group, as did their demand for participation in the plays with dog. They became more colloquial among themselves and with the veterinarian and felt more care with the dog (someone brought prizes for dogs from home). In the interviews, participants reported feeling more pleasure during the hour of therapy with the dog than during other hours, as well as an improvement in their mood when they returned home on the day of the AAA. The behavioural observations the patients made during the sessions without the dog show that they were similar to the initial ones.

Regarding the interaction between oxytocin and serotonin in studies of animal models, it was described that social interaction requires the coordinated activity of oxitocin and serotonin in the nucleus accumbens, similar to in humans. Previous studies have shown that the mechanisms of interaction between oxytocin and serotonin were of particular interest since both molecules are involved in the control of social behaviour. Oxitocin receptors are found on serotonergic cells, while serotonin receptors are found on Oxitocin neurons [69]. Yoshida et al. [70] showed that oxytocin can regulate the release of serotonin and exert anxiolytic effects through the direct activation of the Oxitocin receptor in the raphe nuclei [71]. As already mentioned by Menna et al. 2019 [17], the fifth meeting represents the critical point of the path. One could hypothesize that it represents the point from which the therapeutic alliance is triggered, as was also indicated in many previous reports [72,73,74]. Indeed, these data are consistent with the hypothesis that the initial decrease of 5-HT and OXT levels might represent an adaptation phase to the novelty, rapidly followed by an increase in wellness, characterized by elevated levels of both molecules.

The weak points of the work are due to the limited number of cases (the number of patients and sessions), the scarce knowledge of the cascade effects of these transmitters, which would be the key to understand their role in mediating oxytocinergic functioning, and the absence of well-being measurement by a psychometric test. Further studies concerning socio-demographic factors and other variables should be explored to state the influence of working with a dog on changing the levels of serotonin and oxytocin.

## 5. Conclusions

This was the first study performed on patients on dialysis by monitoring serotonin and oxytocin during a cycle of AAAs with a dog. Based on these preliminary results, we observed that the AAAs with the dog induced a release of serotonin and oxytocin after an initial decrease, which we hypothesized was due to the novelty and the necessary adaptation. A significant increase of serotonin and oxytocin was then observed in the subsequent sessions. However, no significant difference was detected between the beginning and the end of the same session, possibly due to individual variable levels. Finally, the increase of serotonin and oxytocin was not detected in the determinations carried out after the termination of the activities, as serotonin and oxytocin levels settled approximatively on the initial values. Further studies are necessary to deeply evaluate the results obtained.

## Figures and Tables

**Table 1:** Operative sequence of a session of AAA with the dog for dialysis patients.

	Activities	Duration
Step 1	Initial greetings	10′
Step 2	Group Play with the dog: Ball retrieval; Vision pictures of dog’s calming signals, facial expressions of the dog etc. Riddles on animals	20′
Step 3	Interview: Talking with the patient about their hobbies, style life, etc.	20′
Step 4	Final greetings: Dog oral discharge; Hand washing	10′
